# Supplementary material for: The Transcriptional and Translational Landscape of Plant Adaptation to Low Temperatures
Source: Int J Mol Sci. 2025 Sep 4;26(17):8604. doi: 10.3390/ijms26178604 (PMC12428965; doi:10.3390/ijms26178604)
Supplement: Supplementary file 1 [file ijms-26-08604-s001.zip › Figure S5.pdf]

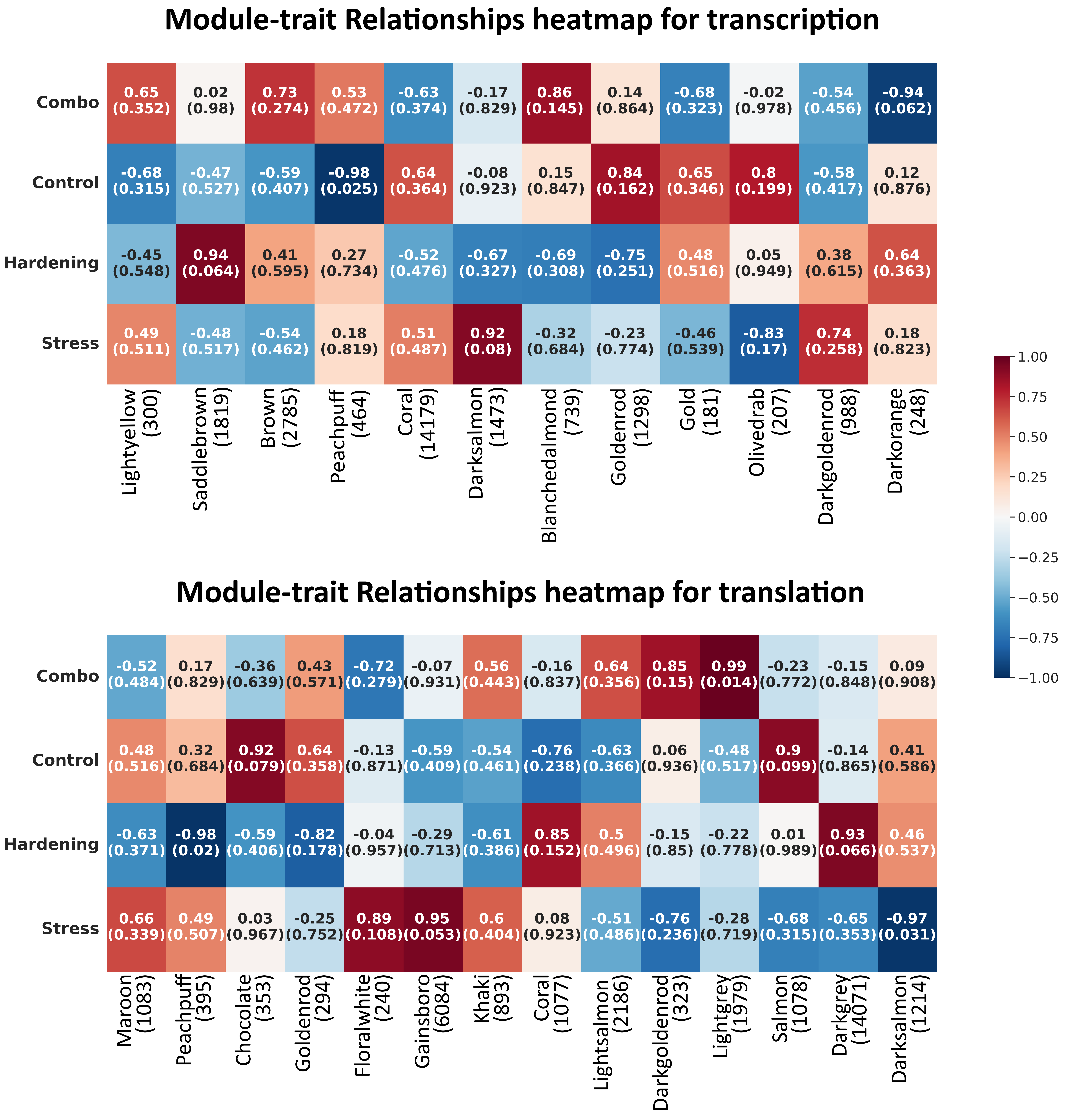
Module-trait Relationships heatmap for transcription
Combo 0.65 (0.352) 0.02 (0.98) 0.73 (0.274) 0.53 (0.472) -0.63 (0.374) -0.17 (0.829) 0.86 (0.145) 0.14 (0.864) -0.68 (0.323) -0.02 (0.978) -0.54 (0.456) -0.94 (0.062)
Control -0.68 (0.315) -0.47 (0.527) -0.59 (0.407) -0.98 (0.025) 0.64 (0.364) -0.08 (0.923) 0.15 (0.847) 0.84 (0.162) 0.65 (0.346) 0.8 (0.199) -0.58 (0.417) 0.12 (0.876)
Hardening -0.45 (0.548) 0.94 (0.064) 0.41 (0.595) 0.27 (0.734) -0.52 (0.476) -0.67 (0.327) -0.69 (0.308) -0.75 (0.251) 0.48 (0.516) 0.05 (0.949) 0.38 (0.615) 0.64 (0.363)
Stress 0.49 (0.511) -0.48 (0.517) -0.54 (0.462) 0.18 (0.819) 0.51 (0.487) 0.92 (0.08) -0.32 (0.684) -0.23 (0.774) -0.46 (0.539) -0.83 (0.17) 0.74 (0.258) 0.18 (0.823)
Lightyellow (300)
Saddlebrown (1819)
Brown (2785)
Peachpuff (464)
Coral (14179)
Darksalmon (1473)
Blanchedalmond (739)
Goldenrod (1298)
Gold (181)
Olivedrab (207)
Darkgoldenrod (988)
Darkorange (248)
Module-trait Relationships heatmap for translation
Combo -0.52 (0.484) 0.17 (0.829) -0.36 (0.639) 0.43 (0.571) -0.72 (0.279) -0.07 (0.931) 0.56 (0.443) -0.16 (0.837) 0.64 (0.356) 0.85 (0.15) 0.99 (0.014) -0.23 (0.772) -0.15 (0.848) 0.09 (0.908)
Control 0.48 (0.516) 0.32 (0.684) 0.92 (0.079) 0.64 (0.358) -0.13 (0.871) -0.59 (0.409) -0.54 (0.461) -0.76 (0.238) -0.63 (0.366) 0.06 (0.936) -0.48 (0.517) 0.9 (0.099) -0.14 (0.865) 0.41 (0.586)
Hardening -0.63 (0.371) -0.98 (0.02) -0.59 (0.406) -0.82 (0.178) -0.04 (0.957) -0.29 (0.713) -0.61 (0.386) 0.85 (0.152) 0.5 (0.496) -0.15 (0.85) -0.22 (0.778) 0.01 (0.989) 0.93 (0.066) 0.46 (0.537)
Stress 0.66 (0.339) 0.49 (0.507) 0.03 (0.967) -0.25 (0.752) 0.89 (0.108) 0.95 (0.053) 0.6 (0.404) 0.08 (0.923) -0.51 (0.486) -0.76 (0.236) -0.28 (0.719) -0.68 (0.315) -0.65 (0.353) -0.97 (0.031)
Maroon (1083)
Peachpuff (395)
Chocolate (353)
Goldenrod (294)
Floralwhite (240)
Gainsboro (6084)
Khaki (893)
Coral (1077)
Lightsalmon (2186)
Darkgoldenrod (323)
Lightgrey (1979)
Salmon (1078)
Darkgrey (14071)
Darksalmon (1214)
1.00
0.75
0.50
0.25
0.00
−0.25
−0.50
−0.75
−1.00
