# Supplementary figures and images for: The Transcriptional and Translational Landscape of Plant Adaptation to Low Temperatures
Source: Int J Mol Sci. 2025 Sep 4;26(17):8604. doi: 10.3390/ijms26178604 (PMC12428965; doi:10.3390/ijms26178604)

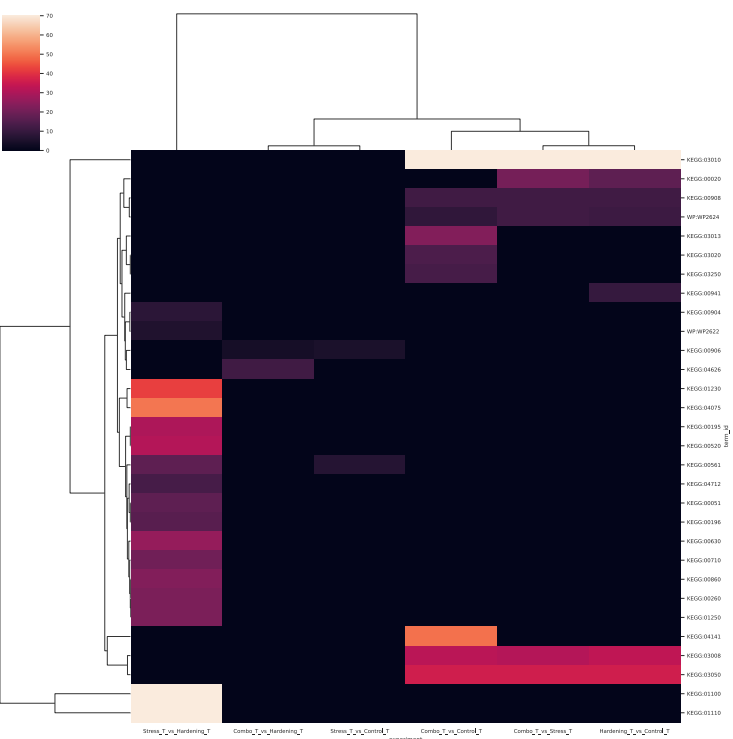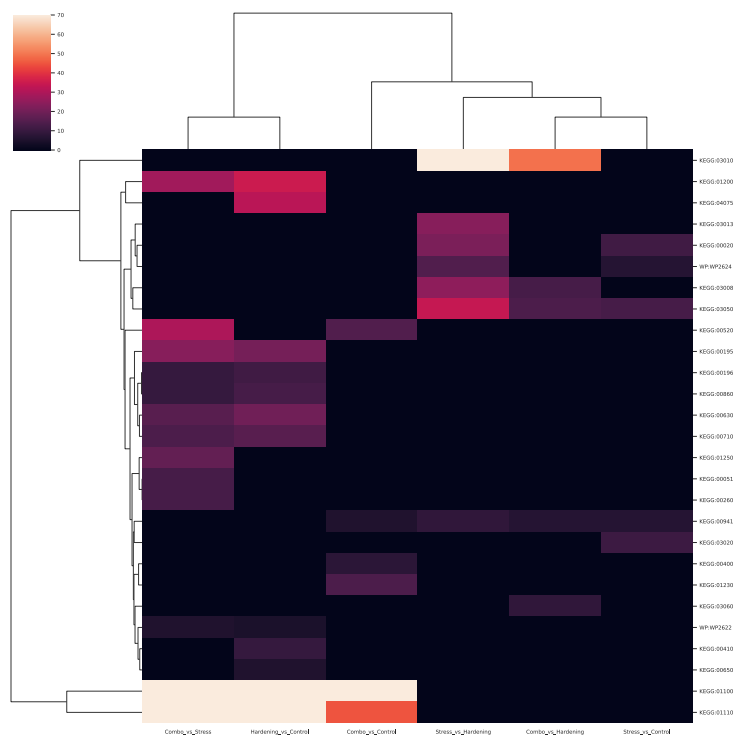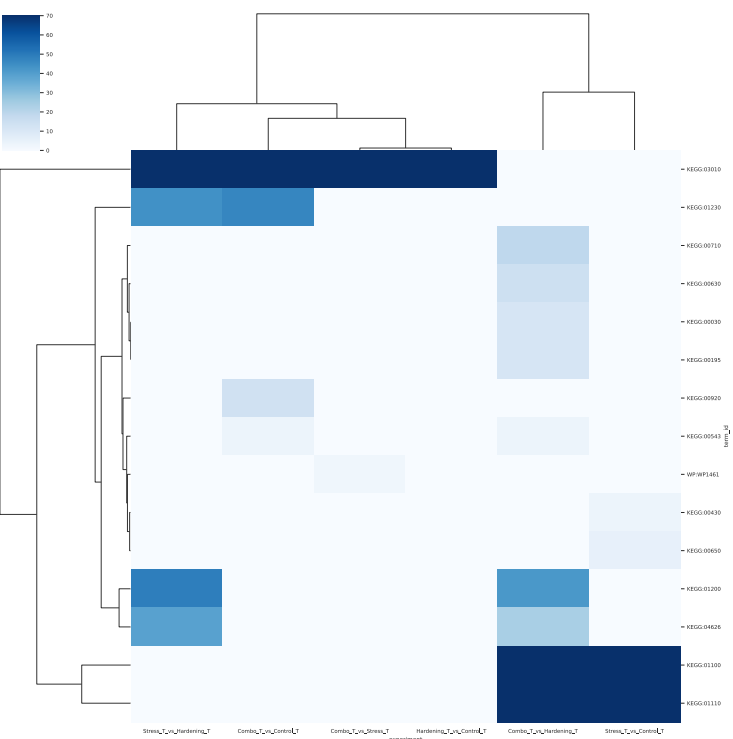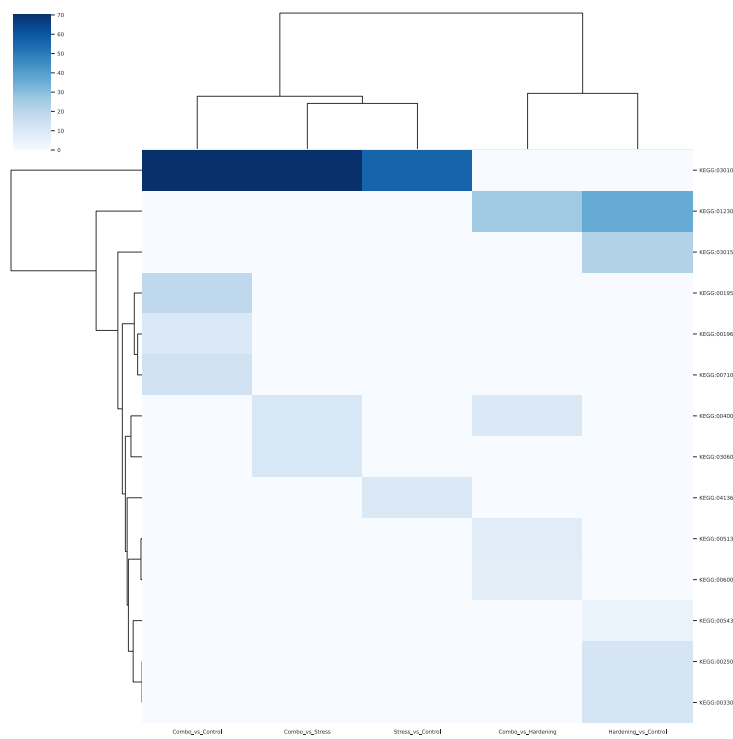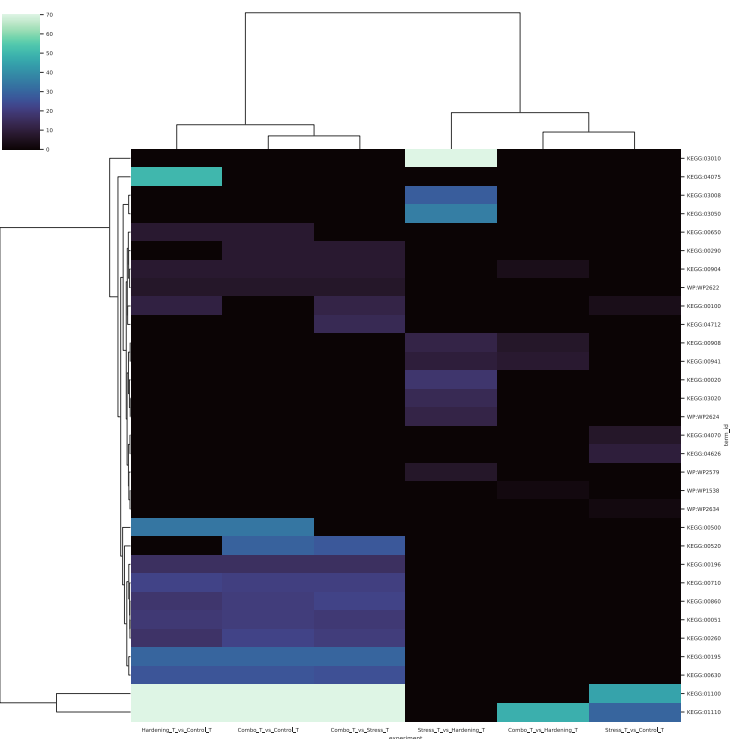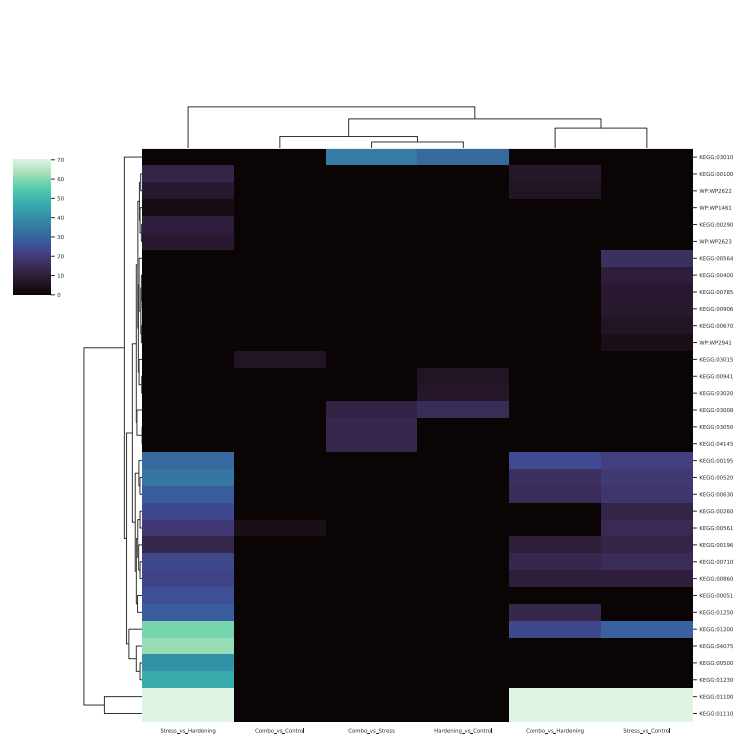

Supplement: Supplementary file 1 [file ijms-26-08604-s001.zip › Figure S1.pdf]

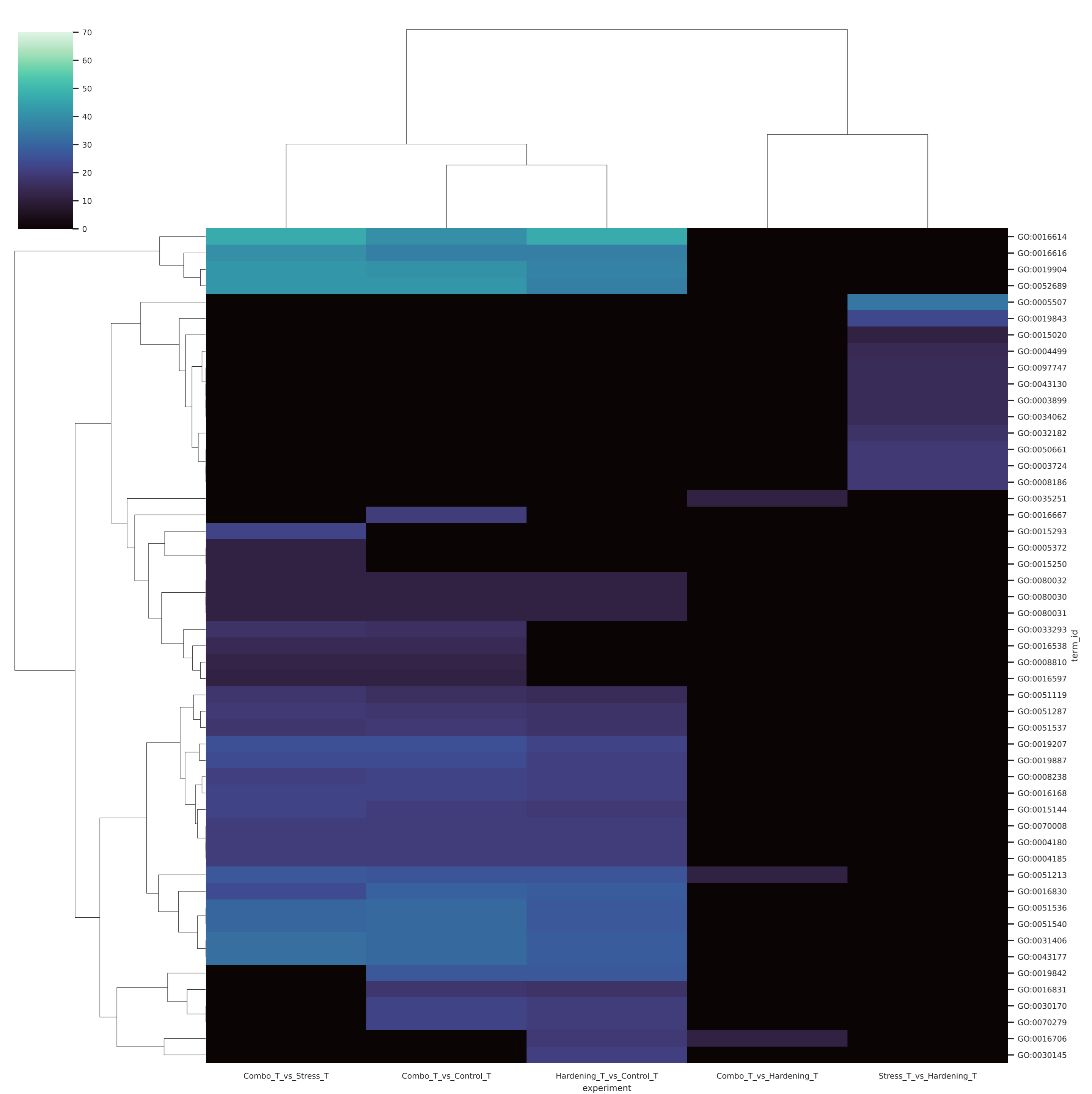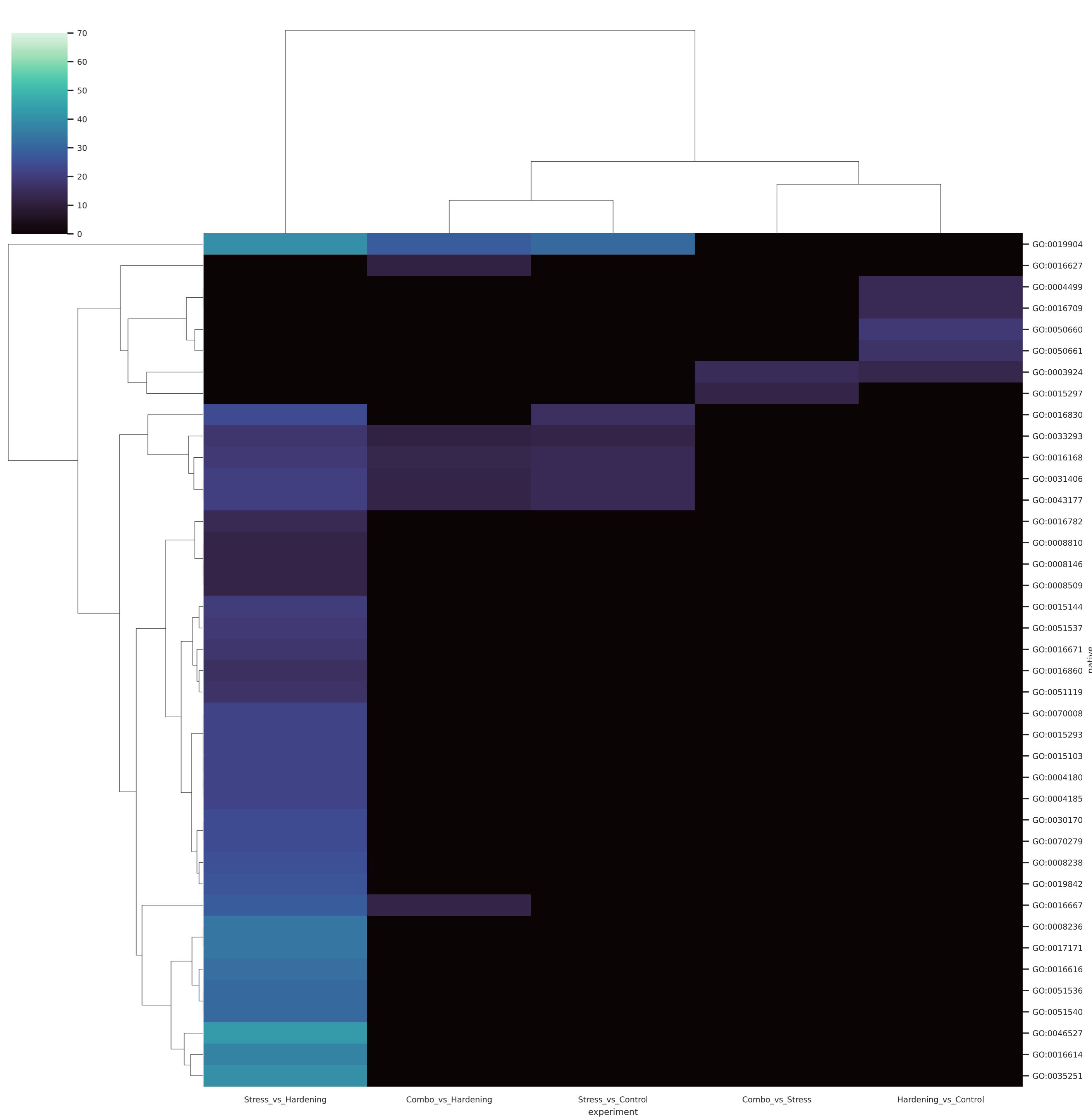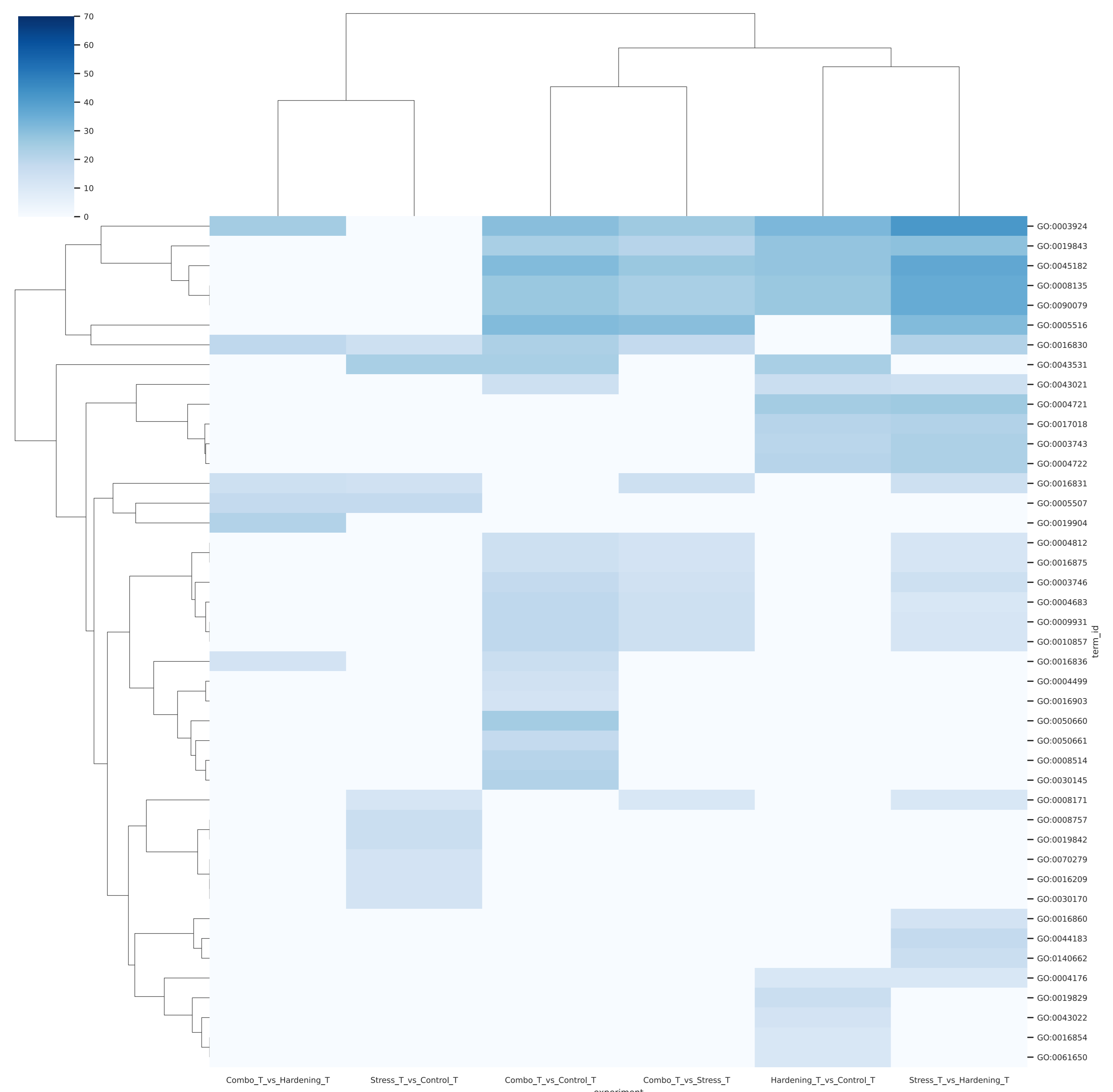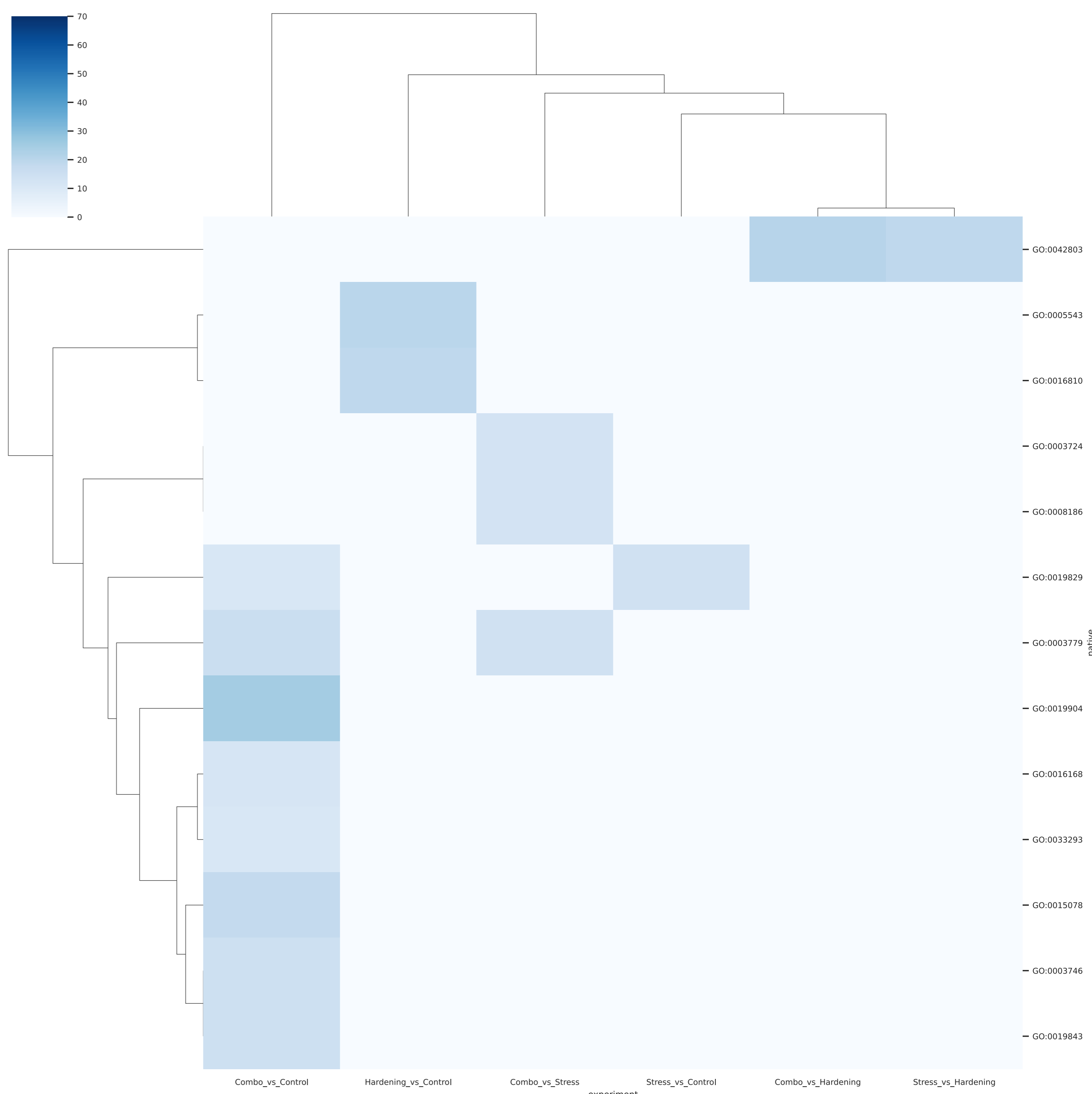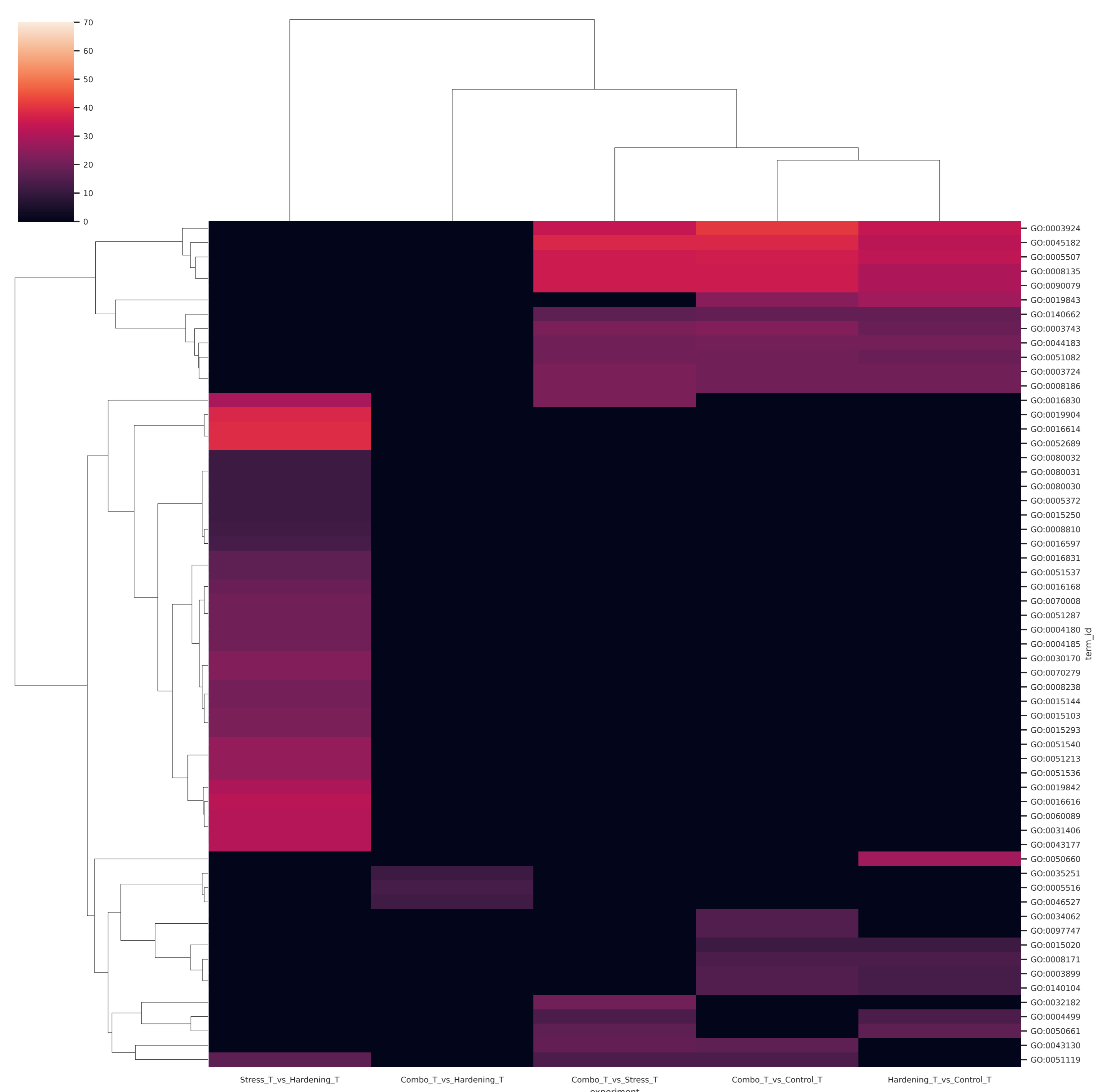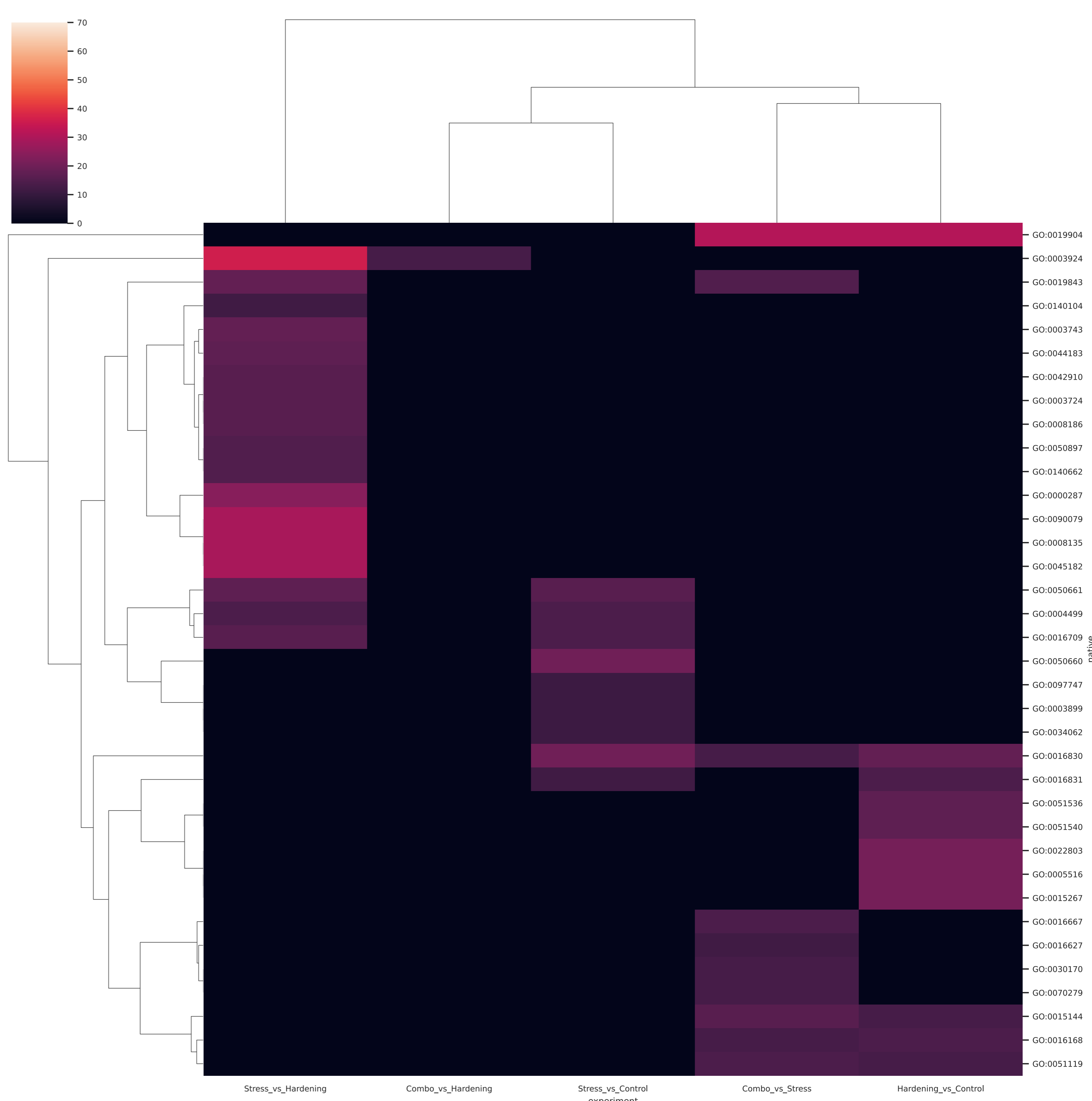

Supplement: Supplementary file 1 [file ijms-26-08604-s001.zip › Figure S2.pdf]

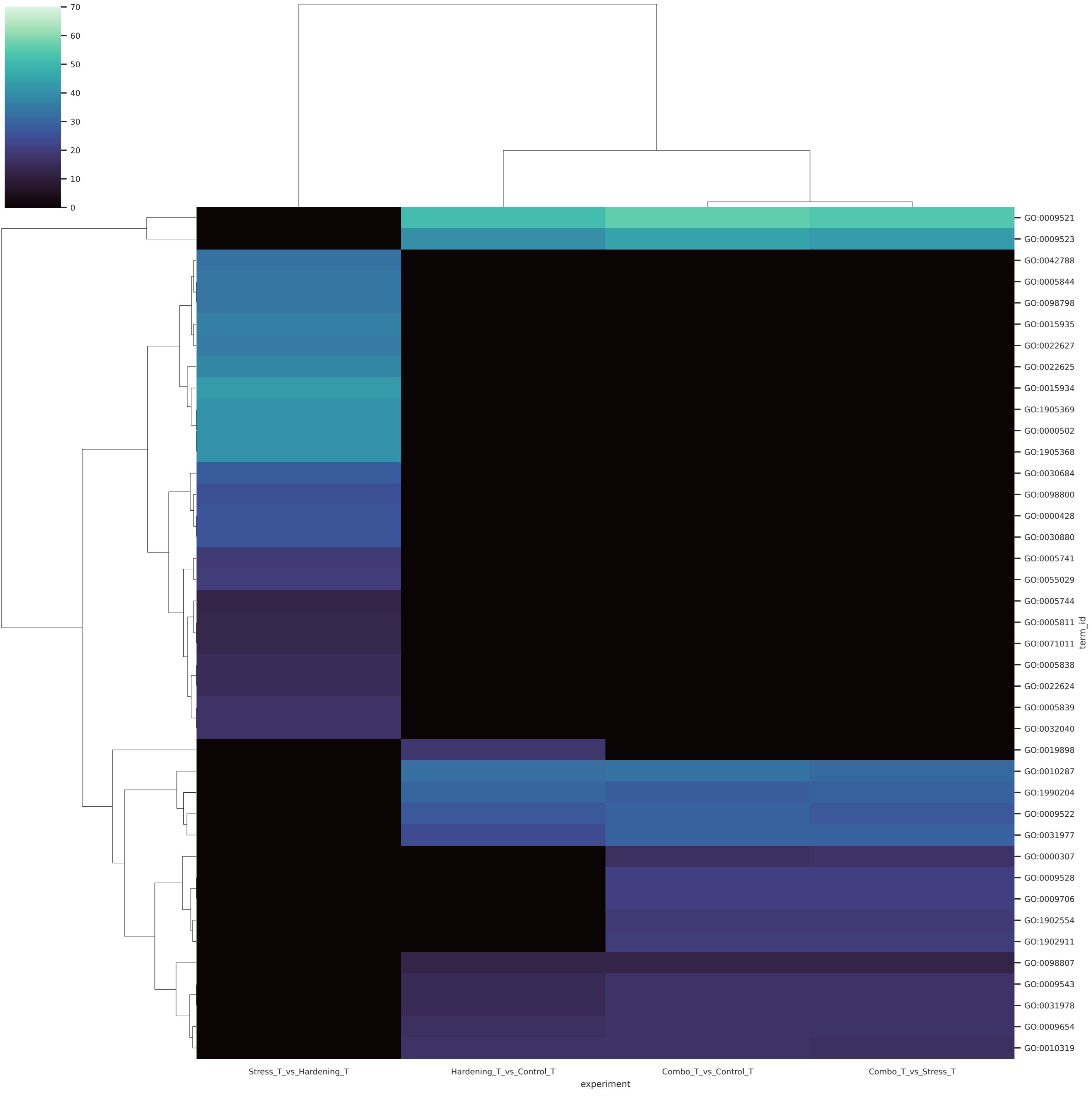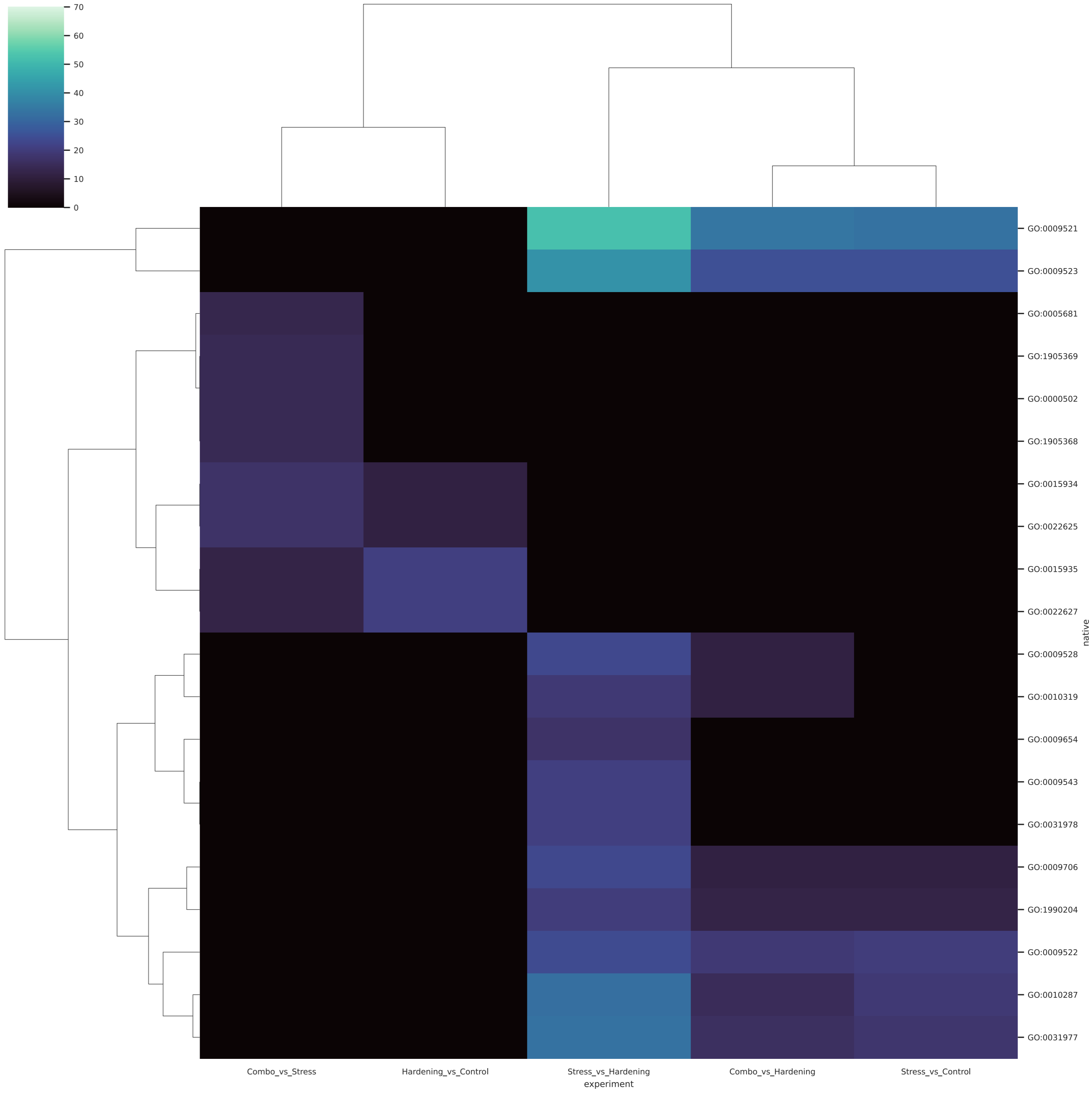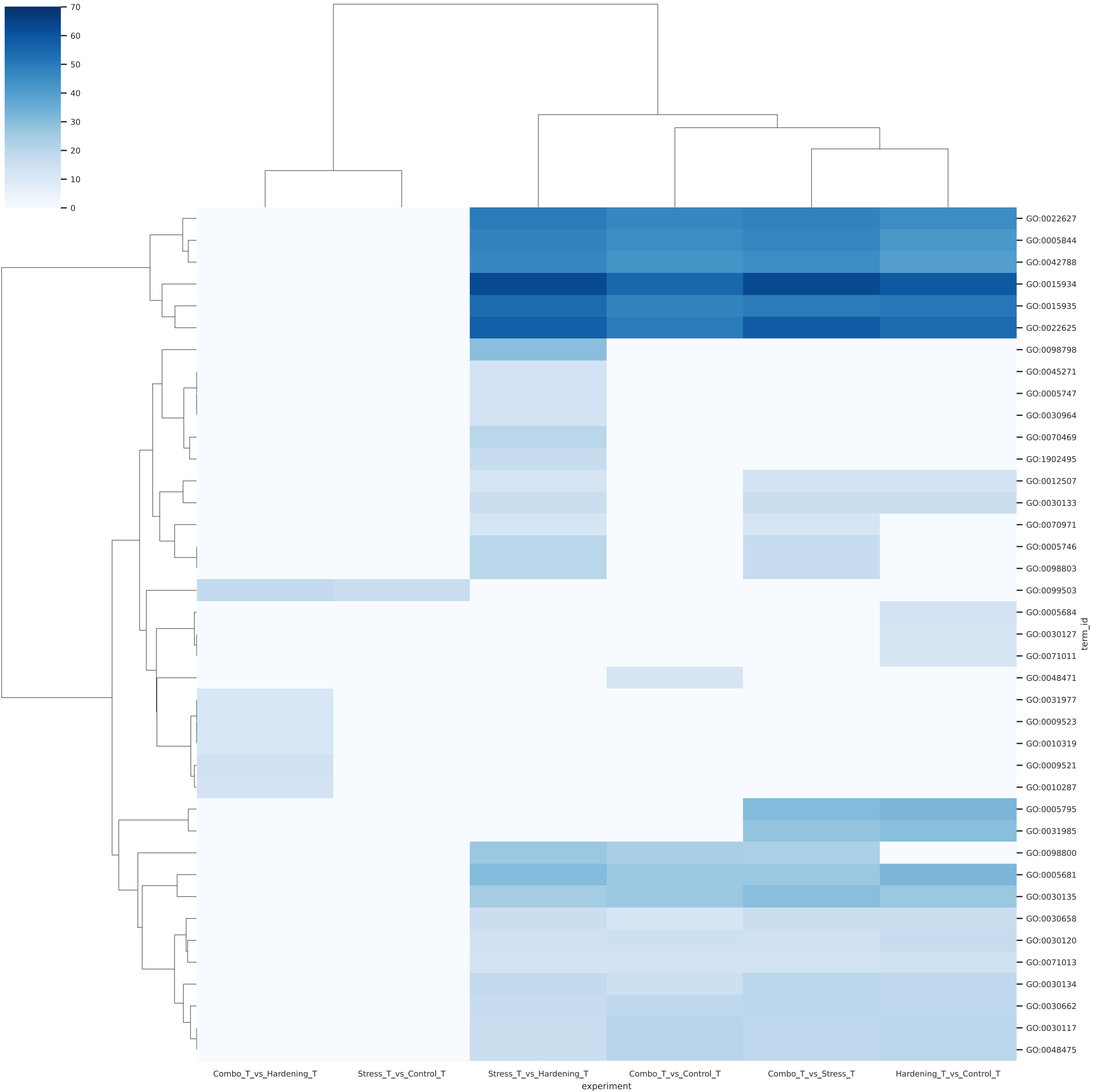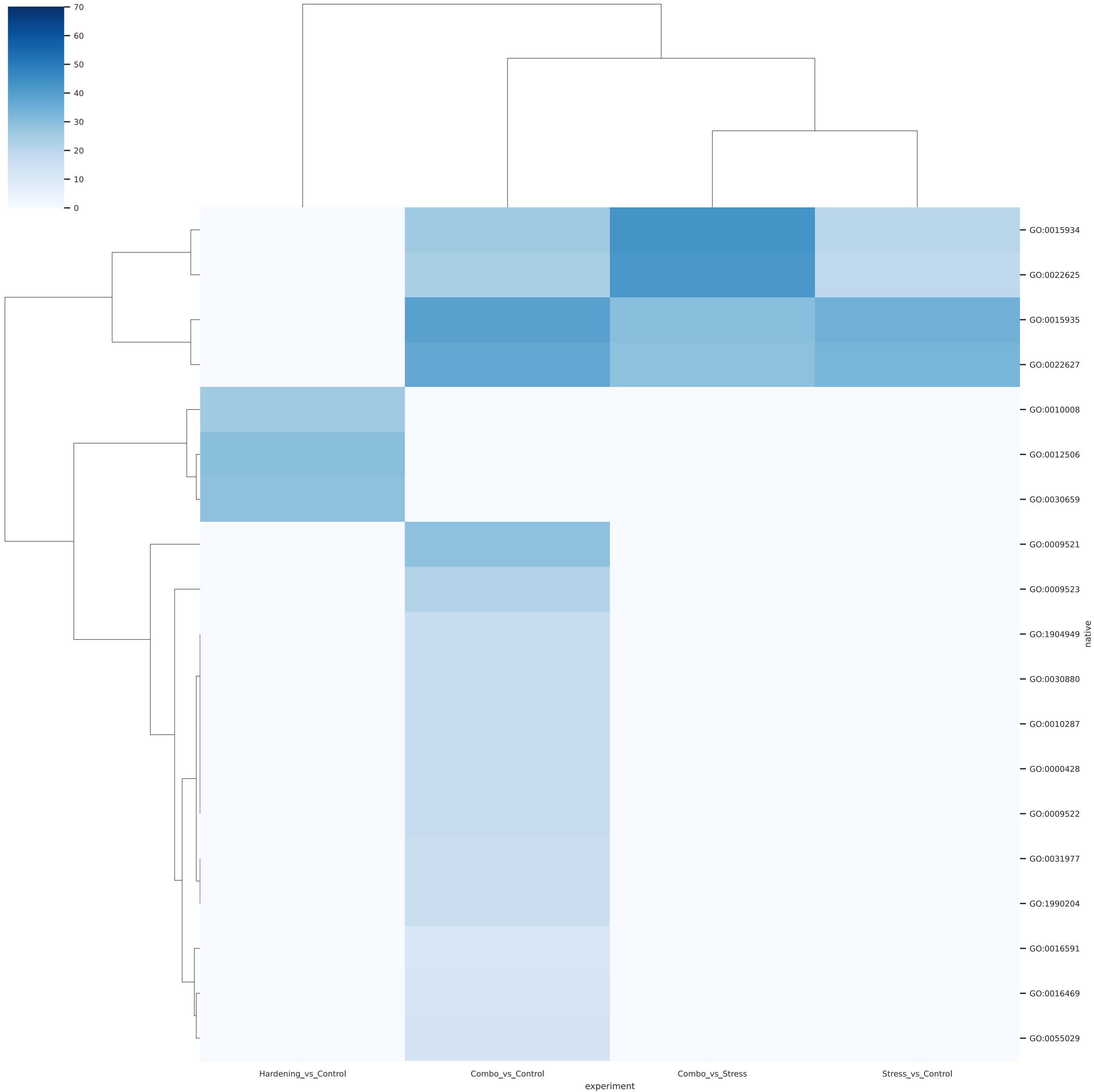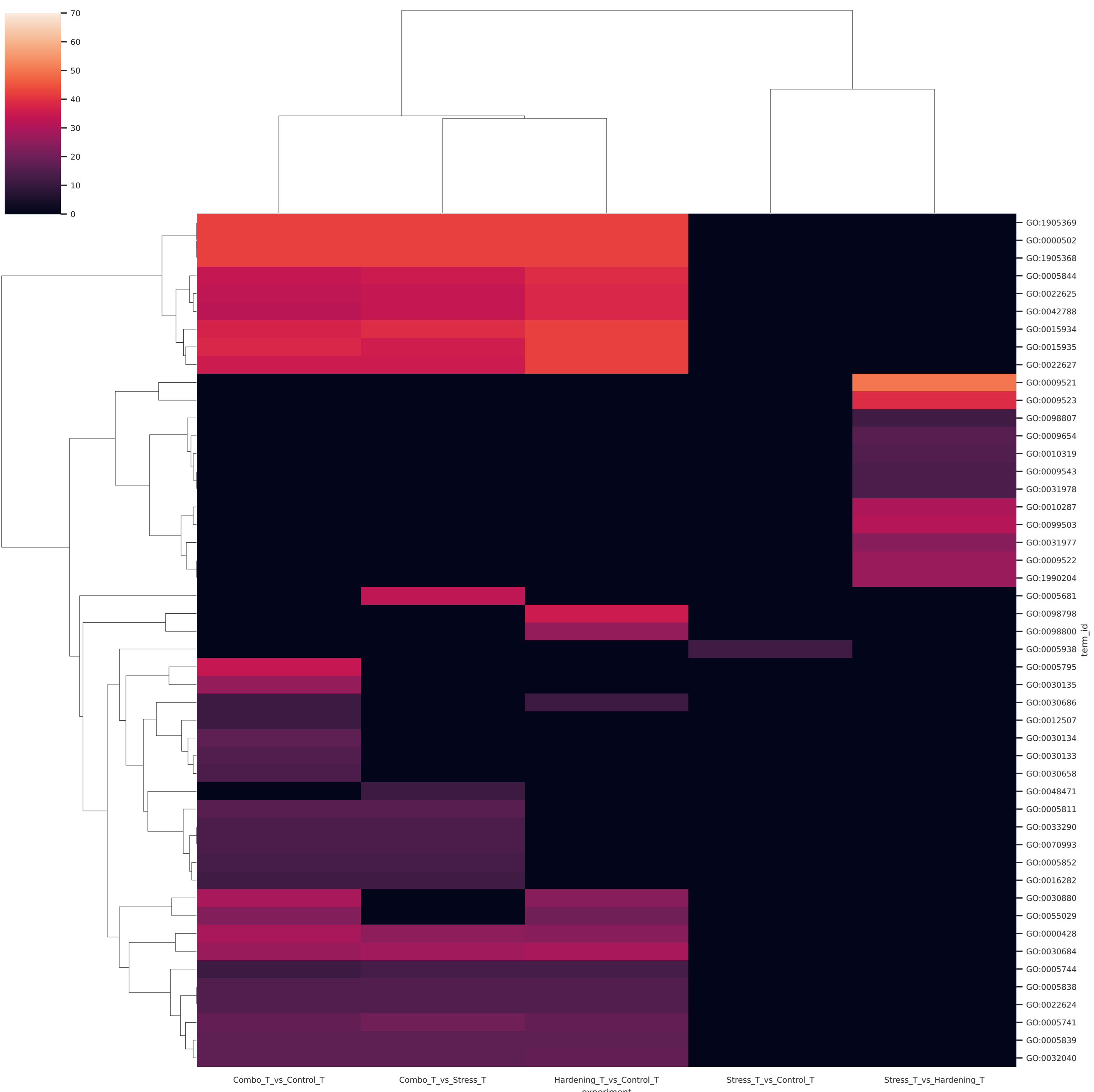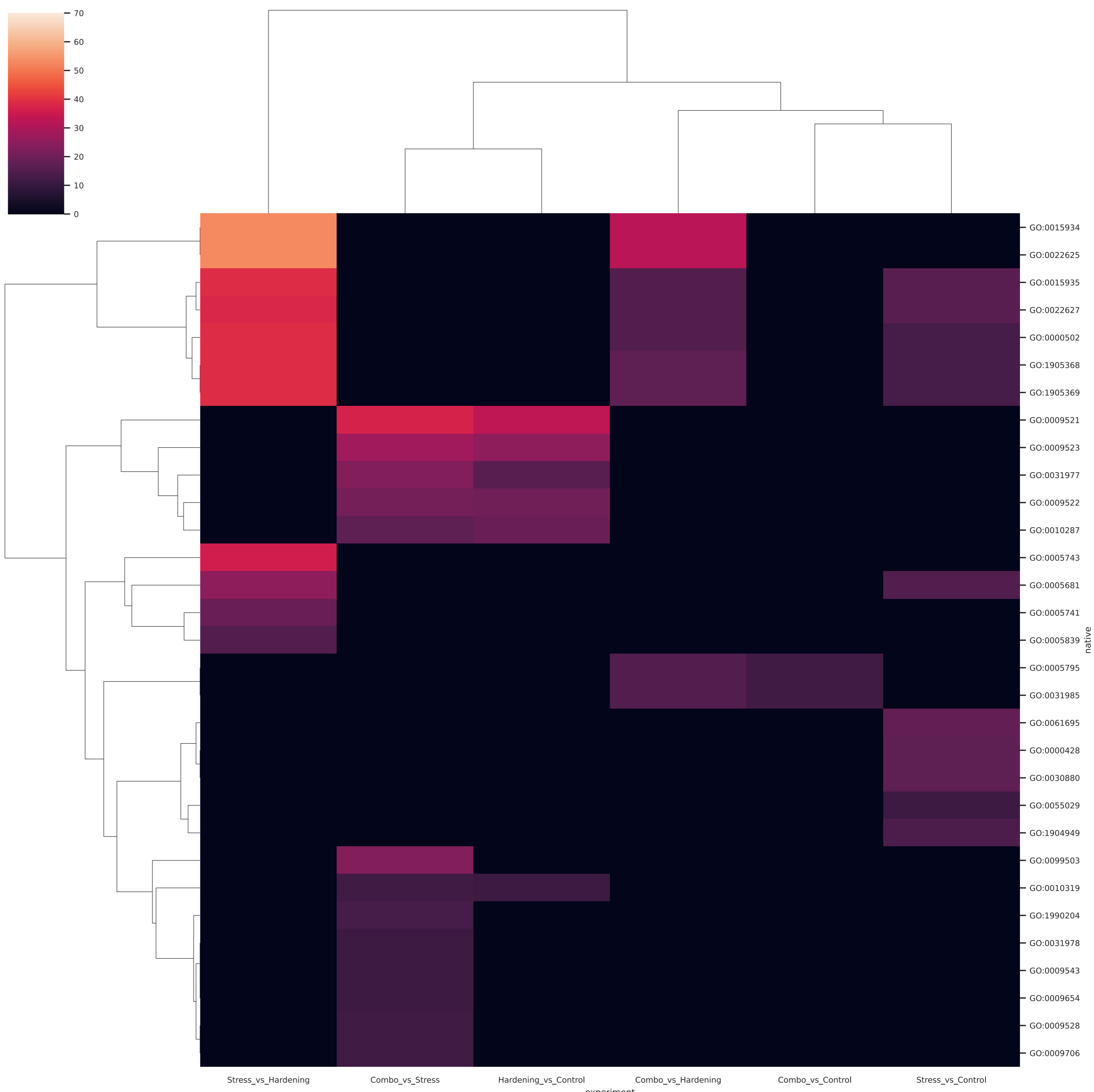

Supplement: Supplementary file 1 [file ijms-26-08604-s001.zip › Figure S3.pdf]

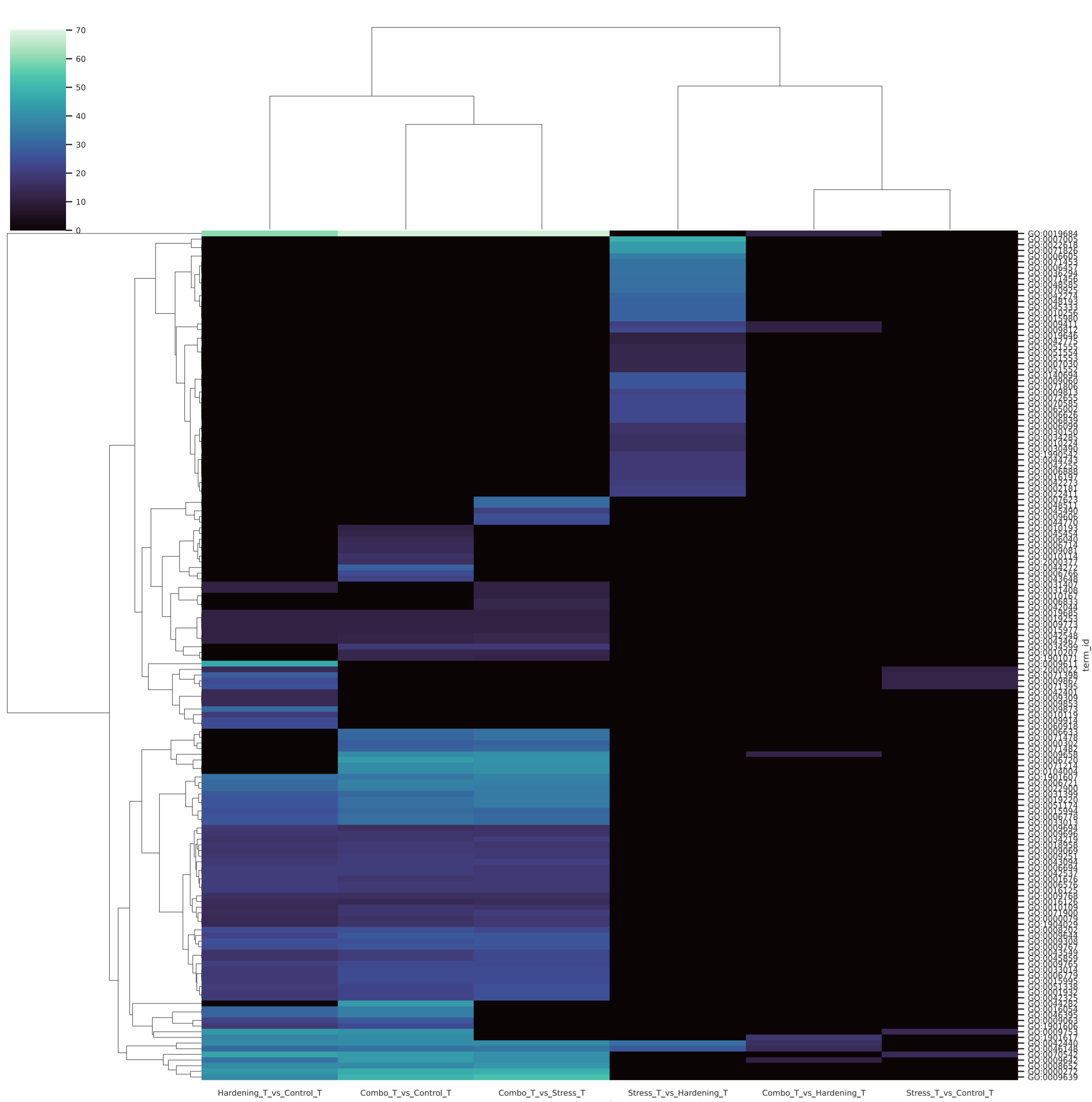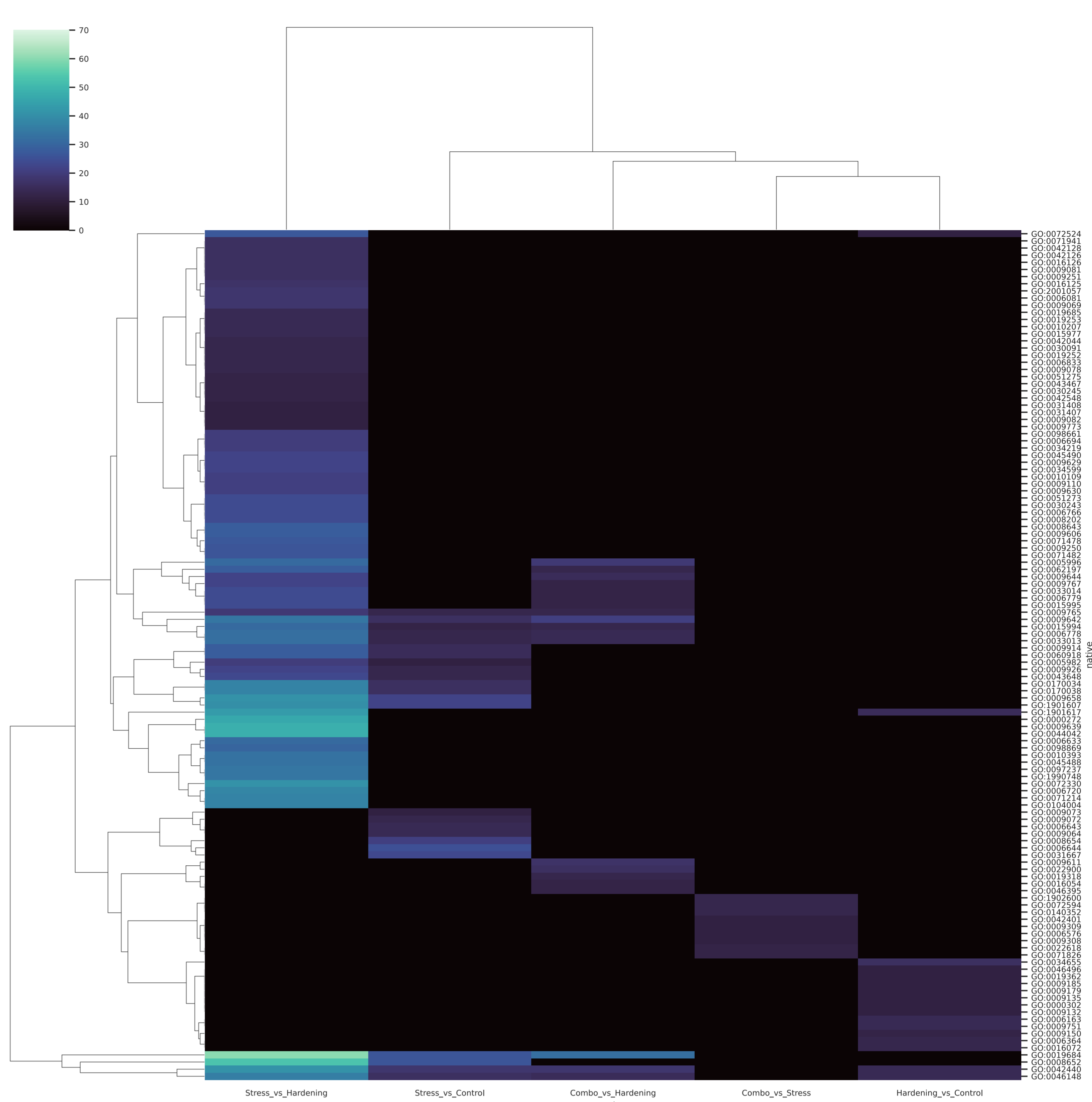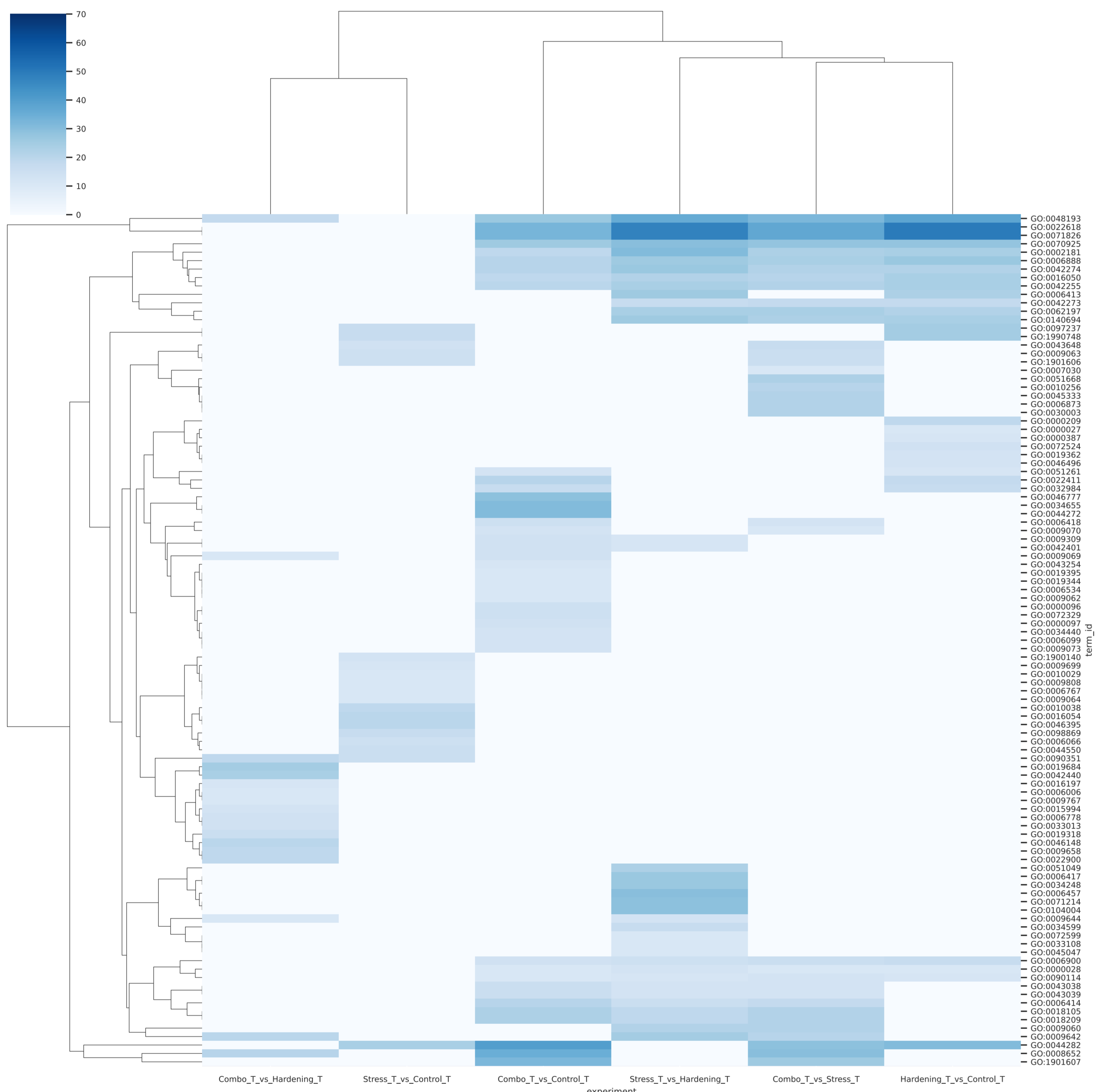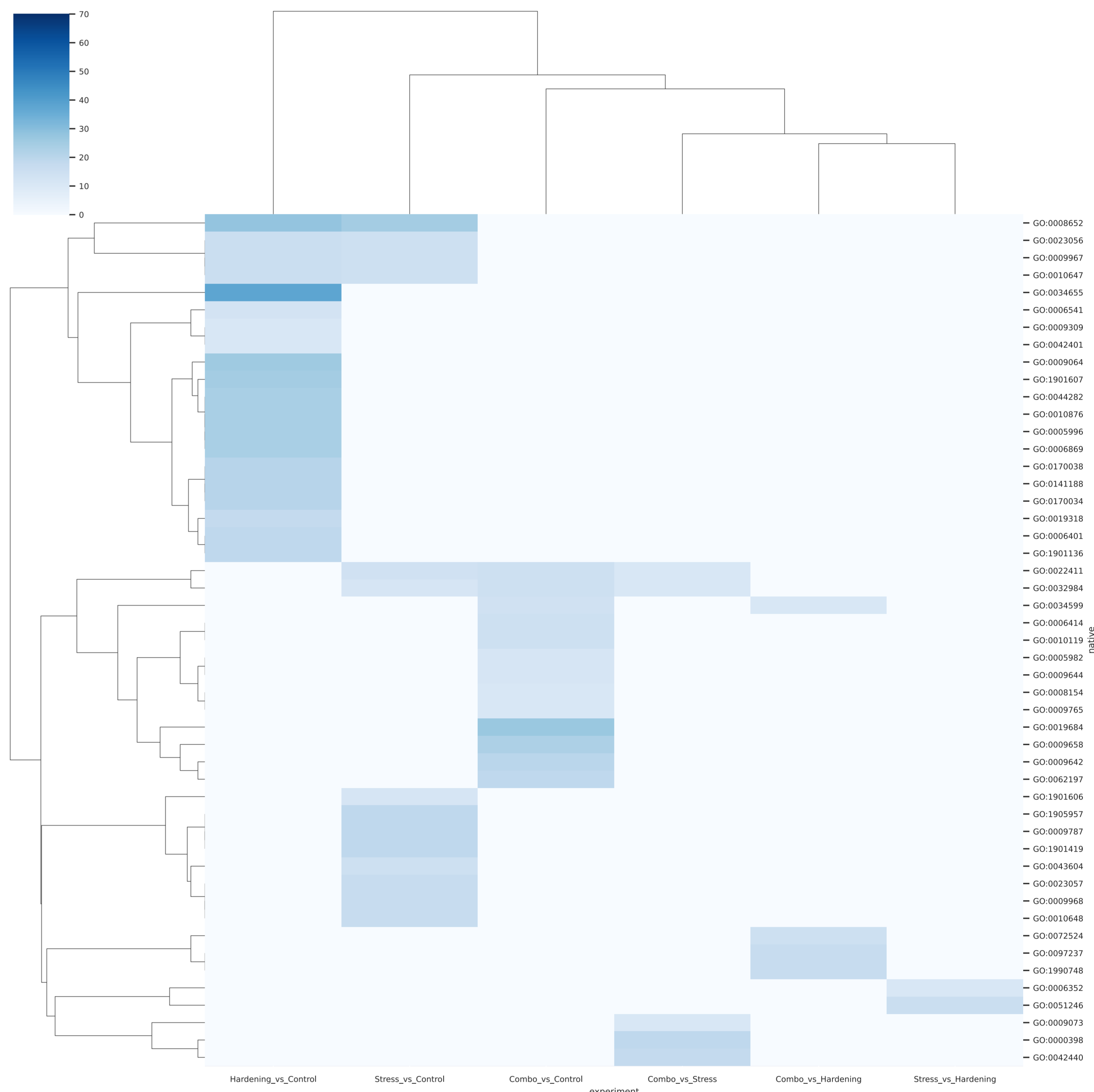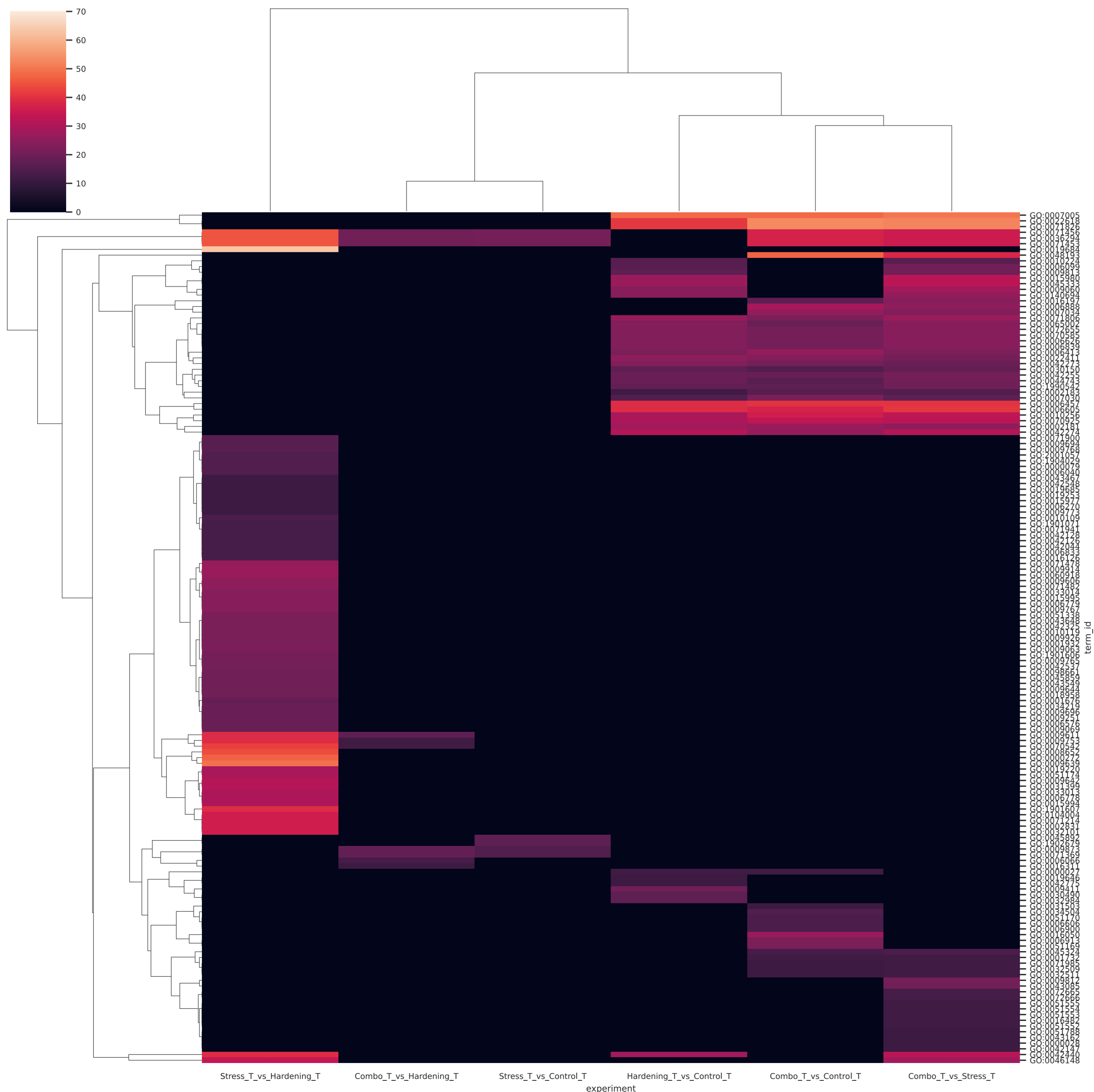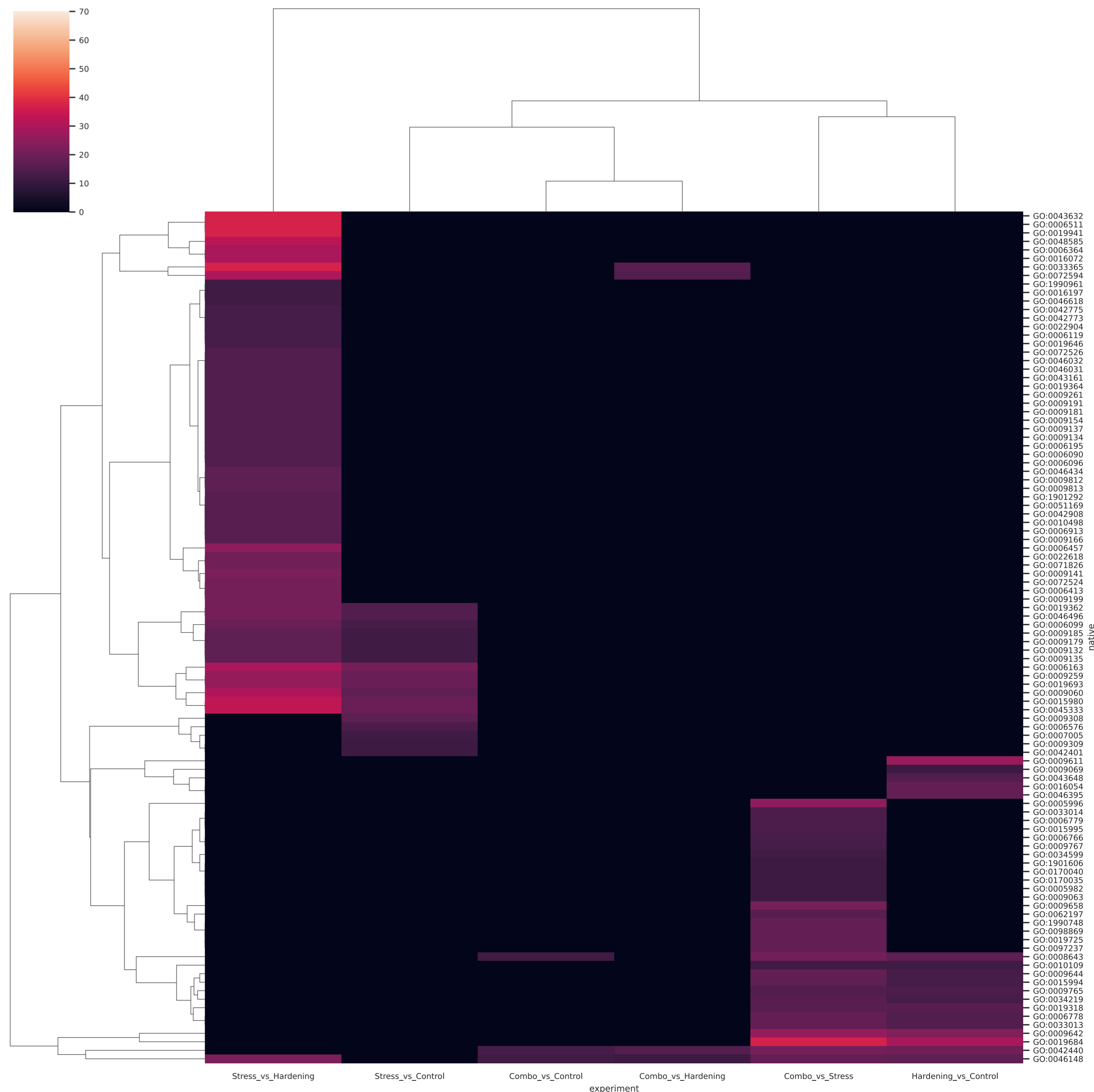

Supplement: Supplementary file 1 [file ijms-26-08604-s001.zip › Figure S4.pdf]

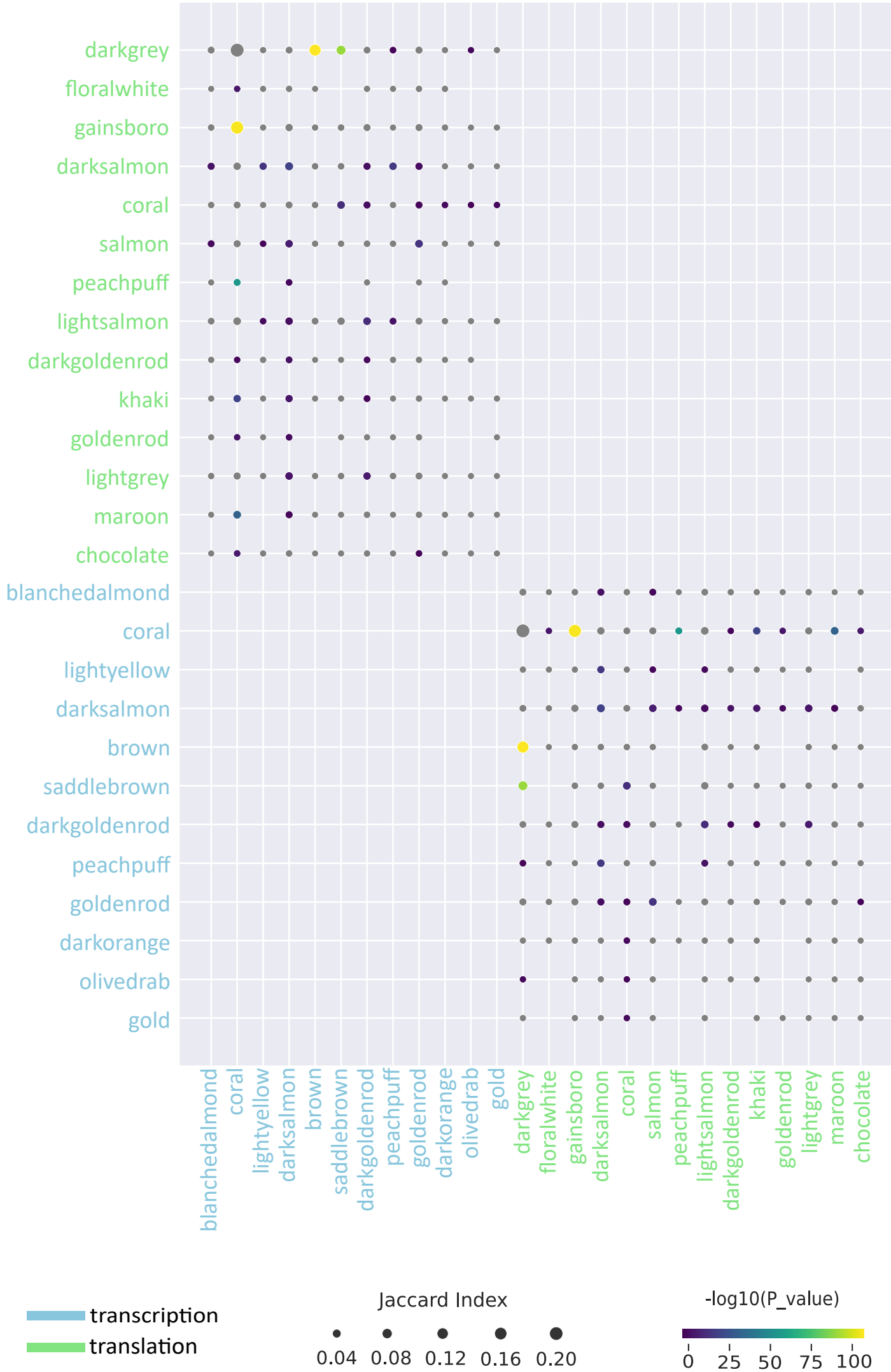

Supplement: Supplementary file 1 [file ijms-26-08604-s001.zip › Figure S6.pdf]
